# Measurement of GSTP1 promoter methylation in body fluids may complement PSA screening: a meta-analysis

**DOI:** 10.1038/bjc.2011.143

**Published:** 2011-06-07

**Authors:** T Wu, E Giovannucci, J Welge, P Mallick, W-Y Tang, S-M Ho

**Affiliations:** 1 Division of Epidemiology and Biostatistics, Department of Environmental Health, University of Cincinnati Medical Center, Cincinnati, OH 45267, USA; 2 Department of Nutrition and Epidemiology, Harvard School of Public Health, Boston, MA 02115, USA; 3 Department of Psychiatry and Behavioral Neuroscience, University of Cincinnati Medical Center, Cincinnati, OH 45267, USA; 4 Division of Environmental Genetics and Molecular Toxicology and Center for Environmental Genetics, University of Cincinnati Medical Center, Cincinnati, OH 45267, USA

**Keywords:** prostate cancer diagnosis, methylation sensitive restriction endonuclease-qPCR, serial testing, primer sequence, methylated and unmethylated sequence, molecular epidemiology, quantitative methylation test

## Abstract

**Background:**

Prostate-specific antigen (PSA) screening has low specificity. Assessment of methylation status in body fluids may complement PSA screening if the test has high specificity.

**Method:**

The purpose of this study was to conduct a meta-analysis of the sensitivity and specificity for prostate cancer detection of glutathione-s-transferase–π (GSTP1) methylation in body fluids (plasma, serum, whole blood, urine, ejaculate, and prostatic secretions). We conducted a comprehensive literature search on Medline (Pubmed). We included studies if they met all four of the following criteria: (1) measurement of DNA methylation in body fluids; (2) a case-control or case-only design; (3) publication in an English journal; and (4) adult subjects. Reviewers conducted data extraction independently using a standardised protocol. Twenty-two studies were finally included in this paper. Primer sequences and methylation method in each study were summarised and evaluated using meta-analyses. This paper represents a unique cross-disciplinary approach to molecular epidemiology.

**Results:**

The pooled specificity of GSTP1 promoter methylation measured in plasma, serum, and urine samples from negative-biopsy controls was 0.89 (95% CI, 0.80–0.95). Stratified analyses consistently showed a high specificity across different sample types and methylation methods (include both primer sequences and location). The pooled sensitivity was 0.52 (95% CI, 0.40–0.64).

**Conclusions:**

The pooled specificity of GSTP1 promoter methylation measures in plasma, serum, and urine was excellent and much higher than the specificity of PSA. The sensitivity of GSTP1 was modest, no higher than that of PSA. These results suggest that measurement of GSTP1 promoter methylation in plasma, serum, or urine samples may complement PSA screening for prostate cancer diagnosis.

Prostate cancer is the most common cancer in men and the second leading cause of cancer-related death in both the United States and Western Europe. With a sensitivity of 80%, prostate-specific antigen (PSA) screening significantly increases the early diagnosis of prostate cancer (Catalona, 1994). However, the specificity of PSA screening is only 20% and may lead to many unnecessary biopsies and overtreatment. A highly specific circulating biomarker (using plasma, serum, or urine samples) that complements the traditional PSA test is therefore in great demand. A specific and non-invasive test would allow patients to avoid the physical pain and discomfort associated with biopsies, and avoid the adverse effects and unnecessary medical spending resulting from overtreatment. A blood draw is already essential for PSA screening and urine samples are easy to obtain; thus, in conjunction with measuring PSA levels, an additional measurement of plasma, serum, or urinary biomarkers does not place any extra burden on patients.

Gene promoter CpG island hypermethylation is one of the earliest somatic genome alterations during the development of several types of cancers. Studies have shown that glutathione-s-transferase—π (GSTP1) promoter hypermethylation is the most common somatic genome alteration during prostate cancer development (Lee *et al*, 1994; Goessl, 2000; Kang *et al*, 2004). If GSTP1 promoter hypermethylation can be detected in body fluids and if it accurately predicts prostate cancer, then this measurement has the potential to complement PSA screening. Research has shown that the prostate, as well as circulating phagocytic cells that have ingested prostate cancer cells, can release DNA into blood circulation (Nakayama *et al*, 2004). DNA can also appear in urine, ejaculates, and prostatic secretions after cells are shed into prostatic ducts. Therefore, detection of methylation status in body fluids (plasma, serum, whole blood, urine, ejaculates, and prostatic secretion) may complement PSA screening if the test has high specificity. Furthermore, although some studies have explored whether GSTP1 promoter hypermethylation in body fluids is associated with the patient's pathological stage, Gleason score, or PSA level, no meta-analysis has been carried out to summarise these results.

The purpose of this study was to conduct a meta-analysis on the sensitivity and specificity of GSTP1 methylation in body fluids on prostate cancer detection. We assessed the usefulness of several types of body fluids, including whole blood, plasma, serum, buffy coat, urine, ejaculates, and prostatic secretions. We also determined whether GSTP1 methylation was correlated with pathological stage, Gleason score, and/or PSA level among the cases.

## Materials and methods

### Study selection

We conducted a comprehensive literature search on Medline (Pubmed) of articles published between 1966 and January 30, 2010. We used the keywords ‘methylation’ and ‘prostate cancer’ in conjunction with any of the following terms: ‘whole blood’, ‘plasma’, ‘serum’, ‘urine’, ‘ejaculate’, and ‘prostate secrete’. Additional studies were found via the reference lists in the identified articles.

**Figure 1:**
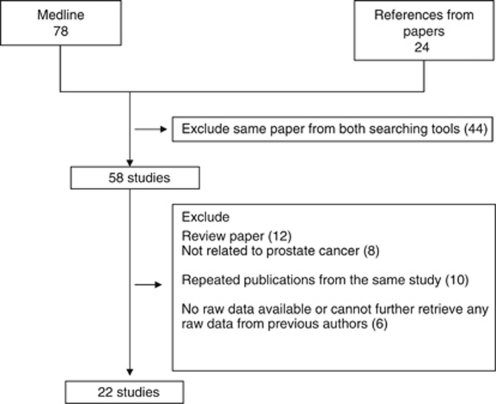
Flow diagram for the selection of studies.

We included the studies that met all four of the following inclusion criteria: (1) measurement of DNA methylation in one of the following body fluids—whole blood, plasma, serum, buffy coat, urine, ejaculates, or prostate secretions; (2) a case-control or case-only study; (3) published in an English language journal; and (4) conducted in adults. We excluded studies that did not test GSTP1 methylation in body fluids. We also excluded studies in which men recently underwent a transurethral resection of the prostate or brachytherapy, or took medications (e.g., Finasteride), because these treatments or medications have the potential to reduce PSA levels. The selection process for studies included in our review is shown in Figure 1. Our search strategy and inclusion/exclusion criteria resulted in a total of 22 articles that were included in the systemic review (Suh *et al*, 2000; Cairns *et al*, 2001; Goessl *et al*, 2001a, 2001b; Jeronimo *et al*, 2002; Gonzalgo *et al*, 2003, 2004; Crocitto *et al*, 2004; Hoque *et al*, 2005; Papadopoulou *et al*, 2006; Rogers *et al*, 2006; Chuang *et al*, 2007; Reibenwein *et al*, 2007; Roupret *et al*, 2007, 2008; Altimari *et al*, 2008; Bastian *et al*, 2008; Bryzgunova *et al*, 2008; Ellinger *et al*, 2008; Woodson *et al*, 2008; Sunami *et al*, 2009). The age range of subjects in the 22 studies was 40–74 years. Details of each study such as mean age of case and control status are included in Supplementary Table 1.

As evaluating the specificity of the methylation test was the major focus of this study, we rigorously classified our controls to remove potential heterogeneity, thereby minimising selection bias. It is well accepted that patients who are referred for biopsies typically have elevated PSA levels, an abnormal digital rectal exam, or related symptoms, and thus are completely different from randomly selected healthy controls. Healthy controls are most likely to introduce bias, resulting in a high specificity. Therefore, among the studies included, we classified controls into two categories: (1) patients who had negative biopsies (10 studies) (Goessl *et al*, 2001a, 2001b; Jeronimo *et al*, 2002; Gonzalgo *et al*, 2003; Crocitto *et al*, 2004; Bastian *et al*, 2008; Ellinger *et al*, 2008; Rossouw *et al*, 2008; Woodson *et al*, 2008; Payne *et al*, 2009) but had other diseases, including benign prostatic hyperplasia, other urologic symptoms (e.g., hematuria), or other types of cancers (e.g., lung cancer or colon cancer); and (2) healthy controls (9 studies) (Papadopoulou *et al*, 2006; Rogers *et al*, 2006; Reibenwein *et al*, 2007; Roupret *et al*, 2007; Altimari *et al*, 2008; Bryzgunova *et al*, 2008; Ellinger *et al*, 2008; Payne *et al*, 2009; Sunami *et al*, 2009). As prostatic intraepithelial neoplasia (PIN) was treated as a case in this study (see below), we excluded the data using PIN as a control in two studies (Goessl *et al*, 2001b; Woodson *et al*, 2008). We excluded Hoque *et al* (2005) from the first category because some of their controls did not have negative biopsies. Although we calculated the specificity separately for the two types of controls, our conclusions were not based on the results generated from healthy controls.

Biopsy-confirmed prostate cancer was treated as a case, except for one study in which biopsy-confirmed PIN was treated as a case. The timing of sample collection from cases varied widely. Some samples were collected prior to biopsies, some after biopsies, some after radical prostatectomy or surgery, and some after hormone therapy. As removing the prostate may influence the likelihood of cancer cells being released into circulation and because hormone treatments may change gene methylation status, we also stratified our analyses by treatment status when samples were collected from cases.

For methylation measurements, we classified them as follows: non-quantitative methylation-specific PCR (N-MSP), quantitative MSP (Q-MSP), methylation-sensitive restriction endonuclease-qPCR (MSRE-qPCR), and bisulfite genomic sequencing. Some methylation assays used a real-time PCR–SYBR Green approach to detect methylated and unmethylated DNA; however, the percentage of methylation is not truly quantified unless methylation and unmethylation control standards are included. Therefore, we considered those assays to be N-MSP. MSRE-qPCR uses a different approach from Q-MSP. It targets the non-bisulfite converted DNA sequence by methylation-sensitive/insensitive endonuclease without using primers specific for methylated and unmethylated sequences; therefore, it is not considered to be Q-MSP in the current manuscript.

As shown in Table 1, 12 studies (Cairns *et al*, 2001; Goessl *et al*, 2001a, 2001b; Jeronimo *et al*, 2002; Gonzalgo *et al*, 2003, 2004; Crocitto *et al*, 2004; Rogers *et al*, 2006; Reibenwein *et al*, 2007; Altimari *et al*, 2008; Roupret *et al*, 2008; Sunami *et al*, 2009) used N-MSP and the same set of methylated/unmethylated primers (∼74 to 170nt) to target the same CpG dinucleotides. Among those using Q-MSP (Chuang *et al*, 2007; Bastian *et al*, 2008; Bryzgunova *et al*, 2008; Ellinger *et al*, 2008), two targeted the region ‘29–168nt’, one targeted ‘179–305nt’, and one ‘7–122nt’. Bastian (2008), Bryzgunova (2008), Chuang (2007), and Ellinger (2008) used MSRE-qPCR or bisulfite sequencing to investigate the methylation status of CpG dinucleotides at the GSTP1 promoter. Although the sequence of primers in these studies (Chuang *et al*, 2007; Bastian *et al*, 2008; Bryzgunova *et al*, 2008; Ellinger *et al*, 2008) did not fall in the ‘+74 to 170 nt’ or ‘+7 to 305 nt’ regions, they were all located at the 5′ promoter region of GSTP1 (−80 to 400 nt from its transcriptional start site).

For our stratified analyses, we classified methylation methods based on their primer sequence, location, and PCR method. We categorised studies using N-MSP as one group and studies using Q-MSP, MSRE-qPCR, or bisulfite sequencing (plus one study (Papadopoulou *et al*, 2006) using gene scan) as another group, because there are not enough sample sizes to further stratify the second group by individual methylation methods.

Among the 22 studies, 11 studies used plasma or serum (Goessl *et al*, 2001a; Jeronimo *et al*, 2002; Papadopoulou *et al*, 2006; Chuang *et al*, 2007; Reibenwein *et al*, 2007; Altimari *et al*, 2008; Bastian *et al*, 2008; Bryzgunova *et al*, 2008; Ellinger *et al*, 2008; Sunami *et al*, 2009), and 11 studies used urine (Cairns *et al*, 2001; Goessl *et al*, 2001a, 2001b; Jeronimo *et al*, 2002; Gonzalgo *et al*, 2003, 2004; Hoque *et al*, 2005; Rogers *et al*, 2006; Roupret *et al*, 2007; Bryzgunova *et al*, 2008; Woodson *et al*, 2008; Payne *et al*, 2009), 1 study used whole blood (Roupret *et al*, 2008), 2 studies used ejaculates (Suh *et al*, 2000; Goessl *et al*, 2001a), and 2 studies used prostate secretions (Crocitto *et al*, 2004; Gonzalgo *et al*, 2004). Some studies collected more than one type of biospecimen.

### Data extraction

Using a standardised data extraction form, two independent investigators (TW and PM) extracted and tabulated all data. Discrepancies were resolved by discussions with other co-authors. We included the author's last name, year of publication, sample size, mean subject age, cancer clinical classification, type of PCR method, and other relevant characteristics of the study population. We extracted the number of positive and negative results among cases and controls. Specifically, the primer location, sequence, and PCR method in each study are summarised in Table 1.

### Statistical analysis

#### Pooled specificity and sensitivity

Sensitivity and specificity estimates from each study were analysed using random-effects models. To assess whether variation in the threshold definition of a positive result produced an association between sensitivity and specificity values across studies, we needed to establish whether there was an association between these parameters. The summary receiving operating characteristic (S-ROC) curve describes the extent of this relationship (Midgette *et al*, 1993). The S-ROC was obtained by estimating the linear regression of the log-odds ratio from each study on the sum of the logits of the true-positive and false-positive rates. When the regression between these quantities is null, independent analyses of pooled sensitivity and specificity using standard methods for binary data are appropriate. In these cases, data were analysed on the log-odds scale (e.g., for specificities, the effect size used was log(Spec/(1-Spec)), with approximate variance (1/R) + (1/(N-R)), where N and R are the number of negative (control) cases and the number of false positives, respectively, in the study. A continuity correction of 0.5 was added to each cell to allow calculations in the presence of zero cell counts.


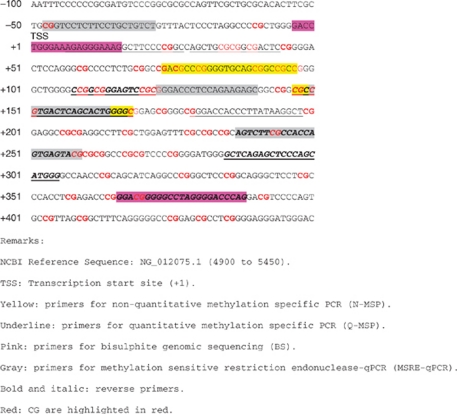


To test for heterogeneity, the Cochrane Q test of heterogeneity (based on deviations of observed log-odds from the common log-odds under a fixed-effect model) was also performed for each analysis.

All analyses were performed using the MIXED procedure in SAS version 9.2. In particular, we observed significant heterogeneity among study outcomes (variation beyond chance expectation), which can complicate the interpretation of findings. However, this is rarely a valid reason for abandoning a meta-analysis altogether. Heterogeneity indicates that a single estimate for the parameter(s) of interest does not hold over all conditions that have been studied and that exploration of which conditions are associated with such variation is warranted. A meta-regression can be used to verify whether any suspected factors may contribute to this heterogeneity. We conducted a meta-regression to examine whether the specificity or sensitivity may be predicted by any of the following factors: age, methylation method, and sample type.

#### Pooled odds ratio (OR)among cases: Methylation associated with pathological stage, Gleason score, and PSA levels

We found that the sensitivity of GSTP1 promoter methylation varied considerably among the studies. We applied the random-effect model to directly analyse the pooled odds ratios of methylation associated with pathological stage, Gleason score, and PSA levels. We adjusted for age, sample type, and methylation method; if none of them appeared to be significant in the model, these variables were removed from the model. Our analyses were limited to samples collected prior to treatment.

## Results

### Individual specificity and pooled specificity of GSTP1 promoter methylation in all studies and in studies using different types of samples

Negative-biopsy controls (16 studies) are shown in Table 2. Most of the GSTP1 specificities were high and above 0.5, although the GSTP1 specificity in one study was below 0.5. The pooled specificity (Table 3) was 0.89 (95% CI, 0.80–0.95), suggesting that the GSTP1 methylation test has a much higher specificity than the PSA test. The *P*-value for heterogeneity was <0.001, indicating significant heterogeneity. Moreover, we performed stratified analyses on the studies according to sample type (plasma/serum or urine) and methylation method (N-MSP or other methods), as shown in Table 3. The specificity was similar between plasma/serum and urine samples regardless of methylation method. Four studies used biospecimens other than plasma/serum or urine. As the sample sizes from these studies were too small to further stratify by methylation method or location of primer, we analysed the pooled specificity of these four studies. The pooled specificity was 0.85 (95% CI, 0.48–0.97)(data not shown). The *P*-value for heterogeneity in each stratum was significant (Table 3).

Meta-regression analyses indicated a nonsignificant inverse association between age and specificity (*β* estimate=−0.22; *P*=0.2). No significant associations were observed between specificity and methylation method or between specificity and sample type.

Finally, the pooled specificity among the studies using healthy controls was high (0.92; 95% CI, 0.81–97), regardless of methylation method and sample type. The *P*-value for heterogeneity was 0.07, suggesting that there may still be variation not attributable to these covariates.

### Individual and pooled sensitivity of GSTP1 promoter methylation

Unlike the relatively high specificity of GSTP1 found among most studies, the sensitivity of GSTP1 varied widely, from 0.05 to 1 (Supplementary Table 2, online only). The overall pooled sensitivity was 0.52 (95% CI, 0.40–0.64) (Table 4). No particular trend was observed among these studies in regard to methylation method or type of specimen (Table 4). The estimated pooled sensitivity for other types of specimens (ejaculate, prostate secretion, and others) was 0.66 (95% CI, 0.35–0.85). As only six studies used other types of specimens, we did not further stratify them by methylation method. The heterogeneity test *P*-values for the overall pooled sensitivity and the individual pooled sensitivity in each stratum were significant.

Upon further exploration, we found that sensitivity was higher in untreated samples (0.63; 95% CI, 0.50–0.75) than in treated samples (0.40; 95% CI, 0.25–0.78), regardless of the specimen type and the methylation method (Table 4). Further stratification by specimen type and methylation method among the treated and untreated groups revealed a higher sensitivity among the untreated (Table 5).

Meta-regression did not reveal any significant associations between sensitivity and age, between sensitivity and methylation method, or between sensitivity and sample type, but the meta-regression did show that sensitivity was significantly lower in the treated samples than in the untreated samples (beta=−1.22, *P*=0.02).

### GSTP1 promoter methylation in relation to pathological stage, Gleason score, and PSA levels among prostate cancer cases

To further evaluate the odds ratios, we converted the pathological stage from an ordinal variable to a binary variable, that is, stages 3–4 *vs* 1–2. Likewise, we categorised Gleason scores as higher than 7 or lower than 7 and grouped PSA levels into higher or lower than 4 ngml^−1^. We limited our analyses to untreated samples and adjusted for methylation method and age; however, if any variable was not significant, it was removed from the model.

**Figure 2:**
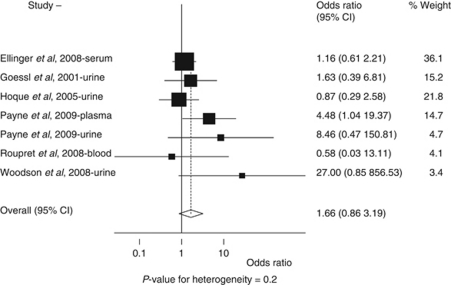
GSTP1 methylation in body fluids collected before treatment and risk of advanced stage of prostate cancer (comparing pathological stages 3–4 to stages 1–2). *P*-value for heterogeneity=0.2.

Six studies (Goessl *et al*, 2001b; Hoque *et al*, 2005; Ellinger *et al*, 2008; Roupret *et al*, 2008; Woodson *et al*, 2008; Payne *et al*, 2009) and additional unpublished data (provided Drs Roupret and James Catto) had sufficient information for analysing the association between gene methylation and pathological stage. We found that GSTP1 promoter methylation increased with prostate cancer pathological stage (stages 3–4 *vs* 1–2), with an odds ratio of 1.66 (95% CI, 0.86–3.19). The *P*-value for heterogeneity was 0.2 (Figure 2). We adjusted for age, sample type, and methylation method in the model; however, as none of these variables were significant, they were removed from the final model. When we excluded the study that used whole blood samples (Roupret *et al*, 2008), the odds ratio slightly increased (OR=1.80; 95% CI, 0.88–3.68; *P* for heterogeneity=0.2).

For similar reasons, we did not adjust for age, methylation method, or sample type for the following analyses. We found that GSTP1 promoter methylation was not associated with Gleason score (pooled odds ratio=1.05, 95% CI, 0.56–1.96; *P*-value for heterogeneity=0.4; 5 studies) (Goessl *et al*, 2001b; Hoque *et al*, 2005; Ellinger *et al*, 2008; Roupret *et al*, 2008; Woodson *et al*, 2008)(additional unpublished data provided by Drs Roupret and James Catto). Furthermore, GSTP1 promoter methylation was not associated with high-PSA levels (odds ratio=0.93, 95% CI, 0.77–1.02; *P*-value for heterogeneity=0.1; 5 studies)(Gonzalgo *et al*, 2004; Hoque *et al*, 2005; Rogers *et al*, 2006; Roupret *et al*, 2007; Woodson *et al*, 2008).

## Discussion

The pooled specificity of GSTP1 was excellent (0. 89, 95% CI, 0.80–0.95) and much higher than the specificity of PSA. The specificity in each subgroup (stratified by sample type and methylation method) remained above 0.86. The sensitivity of GSTP1 was 0.63 (95% CI, 0.50–0.75) for samples collected before treatment and 0.40 (95% CI, 0.25–0.78) for samples collected after treatment; these sensitivities were not higher than the sensitivity of PSA screening. These results suggest that plasma, serum, or urine samples may complement PSA screening for prostate cancer diagnosis, although the positive link between GSTP1 methylation and pathological stage needs to be evaluated in more studies.

Collecting plasma/serum or urine samples is a non-invasive procedure, whereas invasive biopsy procedures may cause pain, anxiety, and increased medical costs. Urine samples were voided urine except the urine samples collected in the following studies: four were collected after a massage (Goessl *et al*, 2001b; Rogers *et al*, 2006; Woodson *et al*, 2008; Payne *et al*, 2009) and one after a biopsy (Rogers *et al*, 2006). High specificity remained even after we excluded the studies with urine collection after a massage.

This study highlights several important issues. First, we identified and systemically evaluated the methylation test at the GSTP1 promoter as an important potential test to complement PSA screening. As a complement rather than a replacement for PSA is needed, a high specificity is more important than a high sensitivity. To combine the strengths of both tests, they should be used sequentially, not simultaneously. The PSA test will be initially used to screen out potential patients, and the GSTP1 methylation test will then be given to those patients who have elevated PSA levels. Only those who have elevated PSA levels, followed by positive results on the GSTP1 methylation test, will undergo further biopsies. With its high specificity, the methylation test will exclude patients unlikely to have PCa but have elevated PSA levels. Using the two tests sequentially will reduce the number of unnecessary biopsies considerably, compared with using the PSA test alone. Serial testing has been used clinically for embolism and diarrhea (Fekety, 1997; Wells *et al*, 2001).

Second, unlike previous studies and reviews, we rigorously evaluated the specificity of GSTP1 by excluding healthy controls. In epidemiological research, we use controls that represent the population from which the cases were derived. As described above, randomly selected healthy controls usually do not have elevated levels of PSA or abnormal urological symptoms; therefore, they cannot represent patients who have high levels of PSA and undergo biopsy tests. Third, no previous studies have systemically evaluated the diagnostic value of measuring GSTP1 promoter methylation in different types of body fluids for prostate cancer diagnosis. This study indicates that the use of plasma/serum or urine samples for prostate cancer diagnosis is an important, non-invasive procedure that can complement PSA screening and minimise unnecessary biopsies.

Future assays that measure DNA methylation at gene promoters need to be standardised, simplified, and evaluated with external quality assurance programmes. Quantitative methods, such as pyrosequencing (<200 bp) and MassArrays (<600 bp), which truly quantify all the DNA methylation in the CpG islands and measure levels of all CpG dinucleotides, are also high-throughput technologies. Therefore, pyrosequencing and MassArrays are considered to be more efficient for validating the DNA methylation of gene promoters.

Of note, Figure 2 indicates that the DNA methylation test in whole blood samples is less sensitive to prostate cancer stages than the same test done in plasma, serum, or urine (Roupret *et al*, 2008). The methylated DNA in plasma, serum, or urine most likely derives from cancer cells whereas the methylated DNA detected in whole blood can be released from white blood cells as well. Therefore, the DNA methylation test in plasma, serum, or urine may be more accurate than the same test applied to whole blood in reflecting the severity of cancer stage. As most prostate cancer cases are detected at an early stage, the current PSA test does not predict specific prostate cancer stages. If GSTP1 methylation in plasma, serum, or urine samples is associated with pathological stage, this test will be even more appealing in addition to its high-specificity feature. More studies are warranted to confirm this finding.

The present study has several limitations. First, the validation assay of gene promoter methylation used in each study was different; some used N-MSP, others used Q-MSP, MSRE-Qpcr, or bisulfite sequencing, adding additional heterogeneity. Gonzalgo *et al*, 2004 (Gonzalgo *et al*, 2004) commented that primers selected at different regions on the same CpG island may have different sensitivities and specificities. Fortunately, the studies included in this paper all chose primers targeting the CpG dinucleotides in the 5′ promoter region starting from −80 to 400 nt from its transcriptional start site; hence findings in these studies are still believed to be appropriate for our analyses, as DNA methylation mostly occurs at the 5′ promoter region. Nevertheless, the specificity of GSTP1 across different methylation methods was consistently higher than the specificity of PSA, even though a wide range of sensitivities for GSTP1 was noted. This indicates the robustness of specificity of the GSTP1 methylation assay. Furthermore, our study did not find an association between sensitivity or specificity and methylation method.

Second, only 15 studies that did not use healthy controls could be used for specificity calculations. However, some of these studies used more than one specimen and gave us additional statistical power. Nevertheless, the evidence is compelling, in that the majority of individual GSTP1 specificities calculated here were higher than 0.8, and the overall pooled specificity was 0.89.

Third, the sample collection time varied widely among the studies. As mentioned above, because of our concern over the influence of treatment, we limited our analysis to untreated cases when analysing the associations between gene methylation and pathological stage and other factors, thereby decreasing our statistical power.

In summary, we summarised primer sequences, nucleotide position, and PCR methods in each study and evaluated them using a meta-analysis, which is a unique approach compared with a traditional review without any statistical analyses. Our study represents a new trend in epidemiology: a cross-disciplinary approach between molecular biology and epidemiology. Measuring DNA methylation at gene promoters has the potential to provide a new generation of biomarkers for prostate cancer diagnosis. Future studies should focus on the following tasks: (1) standardising the primers and the PCR protocols for each target gene; (2) using plasma, serum, or urine samples; (3) using patients with negative biopsies as controls rather than randomly selected healthy controls; and (4) collecting samples from cases before biopsies or at least before treatment to improve sensitivity. These tasks will reduce the heterogeneity among studies, enabling us to conduct an accurate meta-analysis to find a complement for the PSA test. Finally, more studies are needed to examine the association between gene methylation status and the stage and prognosis of prostate cancer. This will help avoid unnecessary treatment of some localised prostate cancers, as prostate cancer therapies are associated with significant adverse effects that impact patients’ health and quality of life.

## Figures and Tables

**Table 1:** Summary of primer sequences used in methylation study of GSTP1

	**Primers sequences** *																				
**Groups**	**Methods**	**Primers location**	**CG's location**	**Forward**										**Reverse**							
Altimari *et al*, 2008	N-MSP^a^	+74 to 170	+80, 90, 94, 147, 150, 168	U	5’-GAT	GTT	TGG	GGT	GTA	GTG	GTT	GTT-3’		5’-CCA	CCC	CAA	TAC	TAA	ATC	ACA	ACA-3’
	N-MSP^a^	+78 to 168	+80, 90, 94, 147, 150, 168	M	5’-TTC	GGG	GTG	TAG	CGG	TCG	TC-3’			5’-GCC	CCA	ATA	CTA	AAT	CAC	GAC	G-3’
Bastian *et al*, 2008	MSRE-qPCR^b^	−4 to +256	+243		5’-GAC	CTG	GGA	AAG	AGG	GAA	AG-3’			5’-ACT	CAC	TGG	TGG	CGA	AGA	CT-3’	
Bryzgunova *et al*, 2008	BS^c^	−4 to +388	43 CpG sites		5’-GAT	TTG	GGA	AAG	AGG	GAA	AGG-3’			5’-CTA	AAA	ACT	CTA	AAC	CCC	ATC	C-3’
Cairns *et al*, 2001	N-MSP	+74 to 170	+80, 90, 94, 147, 150, 168	U	5’-GAT	GTT	TGG	GGT	GTA	GTG	GTT	GTT-3’		5’-CCA	CCC	CAA	TAC	TAA	ATC	ACA	ACA-3’
	N-MSP	+78 to 168	+80, 90, 94, 147, 150, 168	M	5’-TTC	GGG	GTG	TAG	CGC	TCG	TC-3’			5’-GCC	CCA	ATA	CTA	AAT	CAC	GAC	G-3’
Chuang *et al*, 2007	MSRE-qPCR^b^	−48 to +259	−48, +243, +259		5’-CGG	TCC	TCT	TCC	TGC	TGT	CT-3’			5’-CGT	ACT	CAC	TGG	TGG	CGA	AG-3’	
Crocitto *et al*, 2004	N-MSP	+74 to 170	+80, 90, 94, 147, 150, 168	U	5’-GAT	GTT	TGG	GGT	GTA	GTG	GTT	GTT-3’		5’-CCA	CCC	CAA	TAC	TAA	ATC	ACA	ACA-3’
	N-MSP	+78 to 168	+80, 90, 94, 147, 150, 168	M	5’-TTC	GGG	GTG	TAG	CGG	TCG	TC-3’			5’-GCC	CCA	ATA	CTA	AAT	CAC	GAC	G-3’
Ellinger *et al*, 2008	MSRE-qPCR^b^	+123 to 259	+243		5’-GGG	ACC	CTC	CAG	AAG	AGC-3’				5’-ACT	CAC	TGG	TGG	CGA	AGA	CT-3’	
Goessl *et al*, 2001a	N-MSP	+74 to 170	+80, 90, 94, 147, 150, 168	U	5’-GAT	GTT	TGG	GGT	GTA	GTG	GTT	GTT-3’		5’-CCA	CCC	CAA	TAC	TAA	ATC	ACA	ACA-3’
	N-MSP	+78 to 168	+80, 90, 94, 147, 150, 168	M	5’-TTC	GGG	GTG	TAG	CGG	TCG	TC-3’			5’-GCC	CCA	ATA	CTA	AAT	CAC	GAC	G-3’
Goessl *et al*, 2001b	N-MSP	+74 to 170	+80, 90, 94, 147, 150, 168	U	5’-GAT	GTT	TGG	GGT	GTA	GTG	GTT	GTT-3’		5’-CCA	CCC		TAC	TAA	ATC	ACA	ACA-3’
	N-MSP	+78 to 168	+80, 90, 94, 147, 150, 168	M	5’-TTC	GGG	GTG	TAG	CGG	TCG	TC-3’			5’-GCC	CCA	ATA	CTA	AAT	CAC	GAC	G-3’
Gonzalgo *et al*, 2004	N-MSP	+74 to 170	+80, 90, 94, 147, 150, 168	U	5’-GAT	GTT	TGG	GGT	GTA	GTG	GTT	GTT-3’		5’-CCA	CCC	CAA	TAC	TAA	ATC	ACA	ACA-3’
	N-MSP	+78 to 168	+80, 90, 94, 147, 150, 168	M	5’-AGT	TGC	GCG	GCG	ATT	TC-3’				5’-GCC	CCA	ATA	CTA	AAT	CAC	GAC	G-3’
Gonzalgo *et al*, 2003	N-MSP	+74 to 170	+80, 90, 94, 147, 150, 168	U	5’-GAT	GTT	TGG	GGT	GTA	GTG	GTT	GTT-3’		5’-CCA	CCC	CAA	TAC	TAA	ATC	ACA	ACA-3’
	N-MSP	+78 to 168	+80, 90, 94, 147, 150, 168	M	5’-TTC	GGG	GTG	TAG	CGG	TCG	TC-3’			5’-GCC	CCA	ATA	CTA	AAT	CAC	GAC	G-3’
Hoque *et al*, 2005	Q-MSP⁁	+29 to 168	+34, 36, 39, 45, 147, 150, 168	M	5’-AGT	TGC	GCG	GCG	ATT	TC-3’				5’-GCC	CCA	ATA	CTA	AAT	CAC	GAC	G-3’
					Probe	6FAM	CGG	TCG	ACG	TTC	GGG	GTG	TAG	CG-TAMRA							
Jeronimo *et al*, 2002	N-MSP	+74 to 170	+80, 90, 94, 147, 150, 168	U	5’-GAT	GTT	TGG	GGT	GTA	GTG	GTT	GTT-3’		5’-CCA	CCC	CAA	TAC	TAA	ATC	ACA	ACA-3’
	N-MSP	+78 to 168	+80, 90, 94, 147, 150, 168	M	5’-TTC	GGG	GTG	TAG	CGG	TCG	TC-3’			5’-GCC	CCA	ATA	CTA	AAT	CAC	GAC	G-3’
Papadopoulou *et al*, 2006	NA	NA (gene scan)	NA																		
Payne *et al*, 2009	Q-MSP	+179 to 305	+228, 230, 232, 243	M	5’-GGG	ATT	ATT	TTT	ATA	AGG	TT-3’			5’-CCC	ATA	CTA	AAA	ACT	CTA	AAC-3’	
					Probe	5’-AGT	TTC	GTC	GTC	GTA	GTT	TTC	GTT-FL								
					Blockers	5’-CTA	AAC	CCC	ATC	CCC	AAA	AAC	ACA	AAC	CAC	ACA-Cy3					
						LC-	RAD-	TAG	TGA	GTA	CGC	GCG	GTT	CG-PH							
Reibenwein *et al*, 2007	N-MSP	Not published	NA																		
Rogers *et al*, 2006	N-MSP	+74 to 170	+80, 90, 94, 147, 150, 168	U	5’-GAT	GTT	TGG	GGT	GTA	GTG	GTT	GTT-3’		5’-CCA	CCC	CAA	TAC	TAA	ATC	ACA	ACA-3’
		+78 to 168	+80, 90, 94, 147, 150, 168	M	5’-TTC	GGG	GTG	TAG	CGG	TCG	TC-3’			5’-GCC	CCA	ATA	CTA	AAT	CAC	GAC	G-3’
Roupret *et al*, 2008	N-MSP	+74 to 170	+80, 90, 94, 147, 150, 168	U	5’-GAT	GTT	TGG	GGT	GTA	GTG	GTT	GTT-3’		5’-CCA	CCC	CAA	TAC	TAA	ATC	ACA	ACA-3’
	N-MSP	+78 to 168	+80, 90, 94, 147, 150, 168	M	5’-TTC	GGG	GTG	TAG	CGG	TCG	TC-3’			5’-GCC	CCA	ATA	CTA	AAT	CAC	GAC	G-3’
Roupret *et al*, 2007	Q-MSP	+29 to 168	+34, 36, 39, 45, 147, 150, 168	M	5’-AGT	TGC	GCG	GCG	ATT	TC-3’				5’-GCC	CCA	ATA	CTA	AAT	CAC	GAC	G-3’
					Probe	6FAM	CGG	TCG	ACG	TTC	GGG	GTG	TAG	CG-TAMRA							
Suh *et al*, 2000	MSRE-qPCR^b^	+1 to 164	150		5’-TGG	GAA	AGA	GGG	AAA	G-3’				5’-CAG	TGC	TGA	GTC	GC-3’			
Sunami *et al*, 2009	N-MSP	+74 to 170	+80, 90, 94, 147, 150, 168	U	5’-GAT	GTT	TGG	GGT	GTA	GTG	GTT	GTT-3’		5’-CCA	CCC	CAA	TAC	TAA	ATC	ACA	ACA-3’
	N-MSP	+78 to 168	+80, 90, 94, 147, 150, 168	M	5’-TTC	GGG	GTG	TAG	CGG	TCG	TC-3’			5’-GCC	CCA	ATA	CTA	AAT	CAC	GAC	G-3’
Woodson *et al*, 2008	Q-MSP	+7 to 122	+109, 112, 120	M	5’-AGA	GGG	AAA	GGT	TTT	TTC	GGT	T-3’		5’-GCG	AAC	TCC	CGC	CGA-3’			
					Probe	6FAM	TGC	GCG	GCG	ATT	TCG	GG-TAMRA									

*Note:* For Q-MSP, N-MSP and BS, DNA samples are bisulphite-treated prior to PCR. Unmethylated Cytosine (C) is converted to Thymine (T) while methylated-C is protected from bisulphite treatment.

⁁ Q-MSP=quantitative methylation specific PCR. Primers are designed specific for methylated and unmethylated sequences, respectively. An internal probe is included in real-time PCR assay.

Methylation % is quantitatively measured with a methylated DNA control standard.

a N-MSP=non-quantitative methylation specific PCR. Primers are designed specific for methylated and unmethylated sequences.

b MSRE-qPCR=Methylation Sensitive Restriction Endonuclease-qPCR. Primers are designed for non-bisulphite converted DNA sequence. DNA is digested with methylation sensitive restriction endonuclease prior to PCR.

c BS=bisulphite genomic sequencing. Primers are designed at region without any CpG.

NA=Not applicable.

^*^Sequence analysis of GSTP1 is based on NCBI Reference Sequence: NG_012075.1

A total of 43 CpG dinucleotides locating at 5′ promoter region (−80 to +400 nt).

**Table 2:** Specificity from each individual study using only negative biopsies as controls^a^

**Study (15 studies)**	**Specimen type**	**Methylation method**	**Control type**	**No. of negatives**	**No. of total controls**	**Specificity**
Altimari *et al*, 2008	Plasma	N-MSP	BPH	4	5	0.80
Bastian *et al*, 2008	Serum	MSRE-qPCR	No special diagnosis^b^	35	35	1.00
Bryzgunova *et al*, 2008	Serum	BS	BPH	5	5	1.00
	Urine	BS	BPH	5	5	1.00
Chuang *et al*, 2007	Plasma	MSRE-qPCR	BPH	25	27	0.93
Crocitto *et al*, 2004	Prostate secretions	N-MSP	No special diagnosis	19	34	0.56
Ellinger *et al*, 2008	Serum	MSRE-qPCR	BPH	39	42	0.93
Goessl *et al*, 2001a	Buffy coat	N-MSP	BPH	26	26	1.00
	Serum	N-MSP	BPH	22	22	1.00
	Ejaculate	N-MSP	BPH	6	6	1.00
	Urine	N-MSP	BPH	10	10	1.00
Goessl *et al*, 2001b	Urine (after massage)	N-MSP	BPH	44	45	0.98
Gonzalgo *et al*, 2003	Urine	N-MSP	No special diagnosis	11	18	0.61
Jeronimo *et al*, 2002	Urine	N-MSP	BPH	30	31	0.97
	Plasma	N-MSP	BPH	31	31	1.00
	Urine	N-MSP	BPH	30	31	0.97
	Plasma	N-MSP	BPH	31	31	1.00
Payne *et al*, 2009	Plasma	Q-MSP	No special diagnosis	35	51	0.69
	Urine (after massage)	Q-MSP	No special diagnosis	21	51	0.41
Rogers *et al*, 2006	Urine after rectal	N-MSP	No special diagnosis	4	5	0.80
	Urine after biopsy	N-MSP	No special diagnosis	4	5	0.80
Roupret *et al*, 2007	Urine (after message)	Q-MSP	No special diagnosis and BPH	33	38	0.87
Roupret *et al*, 2008	Blood	N-MSP	No special diagnosis	20	22	0.91
Woodson *et al*, 2008	Urine (after massage)	Q-MSP	BPH	68	69	0.99

Abbreviations: BPH=benign prostate hyperplasia; BS=bisulphite genomic sequencing; N-MSP=non-quantitative methylation specific PCR; Q-MSP=quantitative methylation specific PCR; SRE-qPCR=methylation sensitive restriction endonuclease-qPCR.

a We did not ininclude Hoque *et al*, 2005 in which 25 female do not have a prostate biopsy and 25 patients may be healthy.

b Patients with negative biopsies but no special diagnosis.

**Table 3:** Specificity of GSTP1 methylation test in plasma and urine samples

**Number of studies**	**Specimen type**	**Methylation method**	**Pooled specificity**	**95%CI**	***P*-value for heterogeneity**
3	Plasma/serum	N-MSP^a^	0.96	0.78–0.99	*P*<0.001
5	Plasma/serum	Other methods^b^	0.90	0.74–0.96	*P*<0.001
5	Urine	N-MSP	0.90	0.70–0.97	*P*<0.001
4	Urine	Other methods^b^	0.86	0.47–0.98	*P*<0.001
Pooled specificity			0.89	0.80–0.95	*P*<0.001

a N-MSP=non-quantitative methylation specific PCR.

b Other methods include quantitative methylation specific PCR, methylation sensitive restriction endonuclease-qPCR, and bisulphite genomic sequencing.

**Table 4:** Sensitivity of GST-P1 methylation test

**Number of studies**	**Specimen type**	**PCR method/sequence location**	**Pooled sensitivity**	**95%CI**	***P* for heterogeneity**
5	Plasma	N-MSP^a^	0.42	0.18–0.71	<0.001
6	Plasma	Other methods^b^	0.36	0.23–0.52	<0.001
6	Urine	N-MSP	0.45	0.25–0.66	<0.001
5	Urine	Other methods	0.75	0.58–0.86	<0.001
11	Treated^c^	NA	0.40	0.25–0.58	<0.001
12	Untreated^c^	NA	0.63	0.50–0.75	<0.001
Pooled sensitivity			0.52	0.40–0.64	<0.001

a N-MSP=non-quantitative methylation specific PCR.

b Other methods: quantitative methylation specific PCR, methylation sensitive restriction endonuclease-qPCR, and bisulphite genomic sequencing.

c Treated or untreated: represents that the samples were collected either after treatments or before treatments.

**Table 5:** Sensitivity of GST-P1 methylation test stratified by treated and untreated samples

	**Treated^a^**				
**Number of studies**	**Specimen type**	**PCR method/sequence location**	**Pooled sensitivity**	**95%CI**	***P* for heterogeneity**
3	Plasma	N-MSP^b^	0.32	0.13–0.60	<0.001
3	Plasma	Other methods^c^	0.25	0.13–0.41	<0.001
3	Urine	N-MSP	0.42	0.15–0.75	<0.001
1	Urine	Other methods	NA	NA	NA
					
	**Untreated**				
1	Plasma	N-MSP	NA	NA	NA
4	Plasma	Other methods	0.53	0.32–0.73	<0.001
3	Urine	N-MSP	0.49	0.22–0.77	<0.001
4	Urine	Other methods	0.72	0.50–0.86	<0.001

a Treated or untreated: represents that the samples were collected either after treatments or before treatments.

b N-MSP=non-quantitative methylation specific PCR.

c Other methods: quantitative methylation specific PCR, methylation sensitive restriction endonuclease-qPCR, and bisulphite genomic sequencing.
