# Supplementary material for: Measurement of GSTP1 promoter methylation in body fluids may complement PSA screening: a meta-analysis
Source: Br J Cancer. 2011 Jun 7;105(1):65–73. doi: 10.1038/bjc.2011.143 (PMC3137397; doi:10.1038/bjc.2011.143)
Supplement: Supplementary Table 1 and 2 [file bjc2011143x1.doc]

**Supplemental Table 1: Characteristics of cases and controls enrolled in each study**

| **Study** | **County** | **Case** | **Control** | **Mean Age (range) of cases and controls** | **Cancer stage** |
| --- | --- | --- | --- | --- | --- |
| Altimari 2008 | Italy | PCa*, samples collected before treatment^#^ | BPH^ and healthy controls | 65 (50-74) for cases | Pathological stage: T1 to T2  Gleason score ranges: 1-10 |
| Baden 2009 | USA | PCa, samples collected before treatment | BPH | 62 (40-75) for cases and controls | Unknown |
| Bastian 2008 | USA | PCa patients who underwent radical prostatectomy | Patients who had negative biopsies | 58.9 (40-71) for both cases and controls | Gleason score range: 5-10 |
| Bryzgunova 2008 | Russia | PCa, samples collected before treatment | Negative biopsy with BPH diagnosis and healthy controls | 67 for cases, 76.4 for BPH controls and 25.8 for healthy controls | Pathological stage: T1-T3 |
| Cairns 2001 | USA | PCa who underwent radical prostatectomy | No controls | Unknown | Pathological stage: T2 to T3 Gleason score ranges: 5-7. |
| Chuang 2007 | Taiwan | PCa | Negative biopsies with BPH diagnosis | Unknown | Pathological stage: T1 to T3  Gleason score ranges: 5-9 |
| Crocitto 2004 | USA | PCa | Negative biopsies | 65 (48-53) for cases and controls | Gleason score ranges: 5-9 |
| Ellinger 2008 | Germany | PCa, samples collected before radical prostatectomy | BPH | PCa: 65.8 (49-79)  BPH: 68.9 (55-83) | Pathological stage: T1 to T4 Gleason score: 6-8  Invasion status: lymph node invasion, capsular penetration  seminal vesicle invasion, extraprostatic extension and positive surgical margins |
| Goessl 2001 | Germany | PCa | BPH | PCa: 65  BPH:62 | Pathological stage: T1-T4 |
| Goessl 2001b | Germany | PCa, samples collected before any type of treatment | BPH  PIN~ | PCa: 65 (59-81)  PIN: 69 (51-73)  BPH: 62 (47-76) | Pathological stage: T1-T3 |
| Gonzalgo 2003 | USA | PCa and PIN, samples collected before any type of treatment | Negative biopsies | PCa: 68(53-80)  PIN: 56(53-68)  Controls: 63(59-72) | Pathological stage: T2-T3 |
| Gonzalgo 2004 | USA | PCa, samples collected after radical prostatectomy | No controls | PCa: 57(52-61) | Pathological stage: T1-T3  Gleason Score: 5-7 |
| Hoque 2005 | USA | PCa samples collected before curative surgery | Age-matched controls with negative biopsies but have other types of cancer, or urological disorder such as BPH. | PCa: 59 (39-81)  Controls: 57 (28-84) | Pathological stage: T2-T3  Gleason Score: 4-10 |
| Jeronimo 2002 | USA | PCa, samples collected after radical prostatectomy | BPH who underwent transurethral resection of the prostate | PCa:63 (52-74)  BPH: 64 (53-82) | Stage: Unknown |
| Papadopoulou  2006 | Greece | PCa, 12 collected before and 19 after treatments | Healthy controls | Unknown | Unknown |
| Payne 2009 | USA and Germany | PCa, samples collected before any type of treatment | Negative biopsies and healthy controls | PCa: 66 (47-77)  Negative biopsy controls: 62 (48-74)  Young controls:24 (19-30) | Pathologic stage: T1-T4  Gleason Score:4-10 |
| Reibenwein 2006 | Austria | Hormone refractory PCa and early PCa, samples collected after treatments | Healthy controls | Hormone refractory PCa: 67 (50-83)  Early PCa: 70 (58-76)  Healthy controls: age unknown | Pathologic stage: T1-T4  Gleason Score:2-10 |
| Roger 2006 | Germany | PCa, samples collected before any type of treatment | Negative biopsies | PCa: 65 (52-72)  Controls: 61 (46-71) | Pathologic stage: T1-T2  PSA: 0.4-9.6 ng/ml |
| Roupret 2007 | France | PCa, samples collected after radical prostatectomy | Age-matched controls with negative biopsies; some have BPH and some did not | PCa: 63 (56-82)  Controls: 62 (57-79) | Pathologic stage: T2-T3  Gleason Score:4-10 |
| Roupret 2008 | UK | PCa(relapsed)  PCa (Not Relapsed), samples collected before any type of treatment | Normal age-matched control with negative biopsies: PSA above 3 ng/ml | PCa (Relapsed): 75 (65-81)  PCa (Not Relapsed):  73 (58-80),  Matched controls: 62 (57-79) | Pathologic stage  PCa (Relapsed): T2-T4  PCa (Not Relapsed): T2-T4  Gleason Score  PCa (Relapsed): 7-10,  PCa (Not Relapsed):6-9 |
| Suh 2000 | USA | PCa, samples collected before any type of treatment | No controls |  | Gleason Score: 4-6 |
| Sunami 2009 | USA | PCa, samples collected before any type of treatment | Healthy controls | Unknown | Pathological stage: T1-T4  Gleason Score: 5-10 |
| Woodson 2008 | USA | PCa, samples collected before biopsies and any type of treatment | Patients who had negative biopsies but diagnosed as BPH and PIN | PCa:  66 (60-72)  BPH: 61(55-68)  PIN: 69 (54-64) | Pathological stage: T2-T3  Gleason Score: 6-10 |

PCa*: Prostate cancer

BPH^: Benign prostatic hyperplasia

PIN~: Prostate intraepithelial neoplasia

^#^ samples collected before any type of treatment such as surgery, radical prostatectomy, and medicinal treatment.
